# Editorial: Female Hormones: Effect on Musculoskeletal Adaptation and Injury Risk

**DOI:** 10.3389/fphys.2020.00628

**Published:** 2020-06-17

**Authors:** Kirsten Legerlotz, Mette Hansen

**Affiliations:** ^1^Department of Training and Movement Sciences, Humboldt-Universität zu Berlin, Berlin, Germany; ^2^Department of Public Health, Aarhus University, Aarhus, Denmark

**Keywords:** pregnancy, exercise, menstrual cycle, muscle mass, amenorrhea, oral contraceptives, tendon adaptation, ligament injuries

In the context of sport and exercise, sex differences in body composition and performance are more than obvious. However, what we know today about musculoskeletal adaptation and injury risk is predominately applicable to men, as women are vastly underrepresented in research studies. The preference for including male participants may be explained by fear of liability issues should pregnancy occur and concerns about the confounding effects of female hormones on research results (Uhl et al., 2007; Costello et al., 2014), although its precisely the changes in the hormonal profile which have in particular been associated with musculoskeletal injuries (Hewett et al., 2007). As a result, there are still many unanswered questions specifically related to the female athlete, which may put women unnecessarily at risk. With this Research Topic, five papers will add to the literature by providing interesting results on the association of female hormones, musculoskeletal changes and injury risk.

The review by Chidi-Ogbolu and Baar nicely discuss effects of estrogen on all musculoskeletal tissues. Depending on the tissue, injury risk seems differently affected by female hormones. High estrogen levels may increase muscle mass and strength, while at the same time the mechanical properties of tendons and ligaments may be compromised.

Pregnancy, being characterized by rising levels of estrogen and progesterone, has also been thought to be accompanied by augmented connective tissue compliance, which is assumed to lead to an increase in injury risk (Ritchie, 2003). In contrast to non-evidence based common belief, Bey et al. (a) did not detect any change in patellar tendon stiffness during pregnancy and no difference to non-pregnant controls. However, the detected progressive increase in tendon rest length during pregnancy may potentially reduce joint stability, thereby affecting injury risk.

Muscle function is another important factor which may affect injury risk during pregnancy. Indeed, in the study by Bey et al. (b) pregnancy did not increase muscle strength although body mass progressively increased, which possibly increases the risk of falling in the event of balance perturbations. However, Bey et al. (b) detected an increased muscle thickness and pennation angle of the vastus lateralis muscle, pointing toward favorable endocrine conditions promoting muscle growth during pregnancy.

That musculoskeletal adaptation alters depending on hormonal status is underlined by the paper from Dalgaard et al., showing that young oral contraceptive users compared to controls tended to experience a greater increase in muscle mass in response to a supervised progressive resistance exercise training, which was supported by a significantly greater increase in type I muscle fiber cross sectional area. *Post-hoc* analysis indicated that it was users of oral contraceptives containing higher levels of ethinyl estradiol (30 vs. 20 μg/day) who experienced the superior increase in muscle mass.

While the hormonal status affects the response to exercise, exercise itself also elicits a hormonal response. In this context, it was hypothesized that female athletes with secondary functional hypothalamic amenorrhea would have a blunted endocrine response to exercise compared with eumenorrheic athletes. However, Melin et al. could not establish such differences and did not detect any signs of overtraining syndrome in the form of blunted hormonal responses to exercise in any of the investigated women regardless of menstrual status.

In terms of musculoskeletal adaptation and injury risk the compiled studies illustrate, that female hormones may have beneficial and non-beneficial effects at the same time, making it difficult to find a straight answer. Secondly, our view on the effect of female hormones is frequently based on presumptions, highlighting the need for studies in females.

## Author Contributions

KL wrote the draft of the editorial, while MH revised the draft. All authors contributed to the article and approved the submitted version.

## Conflict of Interest

The authors declare that the research was conducted in the absence of any commercial or financial relationships that could be construed as a potential conflict of interest.

## References

[B1] Costello J. T. Bieuzen F. Bleakley C. M. (2014). Where are all the female participants in Sports and Exercise Medicine research? Eur. J. Sport Sci. 14, 847–851. 10.1080/17461391.2014.911354 24766579

[B2] Hewett T. E. Zazulak B. T. Myer G. D. (2007). Effects of the menstrual cycle on anterior cruciate ligament injury risk: a systematic review. Am. J. Sports Med. 35, 659–668. 10.1177/0363546506295699 17293469

[B3] Ritchie J. R. (2003). Orthopedic considerations during pregnancy. Clin. Obstet. Gynecol. 46, 456–466. 10.1097/00003081-200306000-00024 12808395

[B4] Uhl K. Parekh A. Kweder S. (2007). Females in clinical studies: where are we going? Clin. Pharmacol. Ther. 81, 600–602. 10.1038/sj.clpt.6100112 17314925

